# Latent Recycling Potential of Multilayer Films in Austrian Waste Management

**DOI:** 10.3390/polym14081553

**Published:** 2022-04-11

**Authors:** Gerald Koinig, Bettina Rutrecht, Karl Friedrich, Chiara Barretta, Daniel Vollprecht

**Affiliations:** 1 Chair of Waste Processing Technology and Waste Management, Department of Environmental and Energy Process Engineering, Montanuniversitaet Leoben, Franz Josef Straße 18, 8700 Leoben, Austria; bettina.rutrecht@unileoben.ac.at (B.R.); karl.friedrich@unileoben.ac.at (K.F.); daniel.vollprecht@unileoben.ac.at (D.V.); 2 Polymer Competence Center Leoben GmbH, Roseggerstraße 12, 8700 Leoben, Austria; chiara.barretta@pccl.at

**Keywords:** multilayer, monolayer, recycling rate

## Abstract

This work presents a hand sorting trial of Austrian plastic packaging, which showed that according to an extrapolation of the 170,000 t separately collected waste collected in Austria, 30 wt% are flexible 2D plastic packaging. Further, the applications for these materials have been catalogued. The composition of these films was evaluated via Fourier-Transformed Infrared Spectroscopy, which showed that 31% of all films were made of polyethene, 39% of polypropylene, 11% of polyethene–polyethene terephthalate composite, and 8% of a polyethene–polypropylene composite, further resulting in the calculation that of all flexible packaging, 20 wt% are multilayer films. These findings were used to calculate the latent potential for raising the current recycling quota of 25.7% to the mandated rate of 55% in 2030. To this end, scenarios depicting different approaches to sorting and recycling small films were evaluated. It was calculated that through improving the sorting of films the recycling rate could be increased to 35.5%. This approach allows for the recycling of monolayer films by avoiding contamination with foreign materials introduced by multilayer films that impede the recyclates’ mechanical properties. The evaluation showed that sorting multilayer films of this fraction could raise the recycling quota further to 38.9%.

## 1. Introduction

The consumption of plastic packaging has been increasing continuously for years. According to Plastics Europe and Consultic Marketing and consulting carried out in 2016, 49 million tons of oil and gas were used in 2015 to produce plastic, of which around 40% were used for the production of packaging [1,2,3]. The demand for packing that protects products, increases their shelf life, and at the same time stimulates the consumer to buy those products through appealing haptics and optics, contrasts with the need for minimal material use and advantageous product protection while maintaining an ideal proportion of packaging to product mass [4]. Plastic films accomplish this balancing act, reducing the necessary film thickness for achieving essential characteristics such as oxygen barrier function by a factor of one to two orders of magnitude [5]. The annual consumption in Austria of around 69,000 tons of small films, with a surface area of under 1.5 m^2^, per year shows that both consumers and markets have recognised the advantages of thin-film plastic packaging [6].

The consumer usually is unaware that many of these inconspicuous plastic films are high-tech products made up of a composition of different materials, so-called multilayer films [3]. This material composition and the low layer thickness make the foils more desirable than single-layer foils or other packaging materials, and this composition poses a challenge in recycling this fraction [7]. Multilayer films and single-layer films are currently not separated and are recycled together [3]. The problem here is that multilayer films with a composition that deviates from the target material can cause complications in film recycling since this requires single-layer films. Introducing multilayer films to a monolayer fraction diminishes the mechanical properties of the recyclates because of impurities introduced by the multilayer material, increasing degradation of the recyclates [8]. This deterioration reduces the possibilities of recycling for these fractions and only leaves two primary avenues for further use. One is downcycling products for mechanically untaxing applications, and the other is the use as refuse derived fuel for co-incineration. Either approach prohibits recycling into high-value products [9]. Therefore, each input stream of polymers for recycling must be monitored to ensure compatible quality marks, such as melt flow rate, degree of crystallinity, degree of degradation and level, and type of low molecular weight compounds. In particular, polymer mixes in the remelting process lead to problems and quality losses in the end products due to the different melting temperatures of the polymers. Additives used can noticeably change the separation properties of the plastics, e.g., the density, and thus impair the sorting process and the recycling process [10]. Understanding the waste stream’s composition is a prerequisite for creating monitoring systems for this purpose.

The current approach of energy recovery from plastic films wastes valuable resources, and is increasingly problematic as EU guidelines dictate an increase in the recycling rate and demand that 50% of all lightweight packaging be recycled in a cost-efficient and sustainable manner [11]. Achieving the goals set by these new guidelines demand incorporating new technologies to separate monolayer and multilayer films, which could reduce CO**_2_** output and increase the recycling rate.

The basis for these technologies is the knowledge about the composition of two-dimensional post-consumer waste (PCW). Previous studies have decomposed the PCW according to material classes [6], evaluated the composition of waste plastics in the feed of Austrian waste-to-energy facilities [12], or provided an in-depth analysis of plastic flows and stocks in Austria [6].

These analyses of plastic flows and stock showed that the small film fraction sums up to 69,000 tons, equivalent to a share of 40 wt% 2D material in separately collected waste (SCW) and 23 wt% of the total waste packaging plastics input in Austria. Within an extended packaging market, considering the whole of Europe, flexible packaging accounts for 26.1% of all plastic packaging [9]. Given the structure of the plastic packaging films, around 17% of all produced packaging in 2010 films worldwide were multilayer material [7]. 

Van Eygen et al. showed in 2018 that 24 wt% of this small film fraction, consisting of mono- and multilayer materials, are subject to a mechanical recycling process, while 76 wt% of mono- and multilayer films are incinerated or co-incinerated [6]. 

This article aims to enhance the existing findings by evaluating the composition of two-dimensional PCW regarding the amount of multilayer and monolayer materials in the current material stream by quantitatively and qualitatively analysing samples taken from Austrian waste streams. This evaluation will determine the occurrence of multilayer films in the post-consumer waste stream, its material composition and its utilisation options. Future applications for improved recycling technologies for 2D PCW are derived based on the performed hand sorting trials. This comparison of future scenarios for small films recycling is achieved by comparing different scenarios concerning technological developments in recycling small films and their implementations in the Austrian waste management sector regarding their potential to increase the current recycling efficiency.

## 2. Materials and Methods

### 2.1. Sample Description

**Figure 1:**
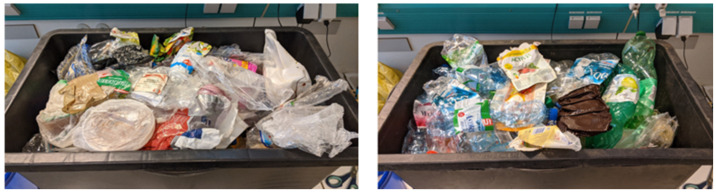
Exemplary manually pre-sorted bulky three-dimensional plastic packaging waste. **Left**: Two-dimensional flexible plastic waste. **Right**: Three-dimensional solid plastic waste.

The samples were taken from the waste collection system “yellow bag”, the collection system for lightweight plastic packaging in rural areas of Austria, during the first quarter of 2021. The waste is collected in yellow bags with a volume of 110 litres. Collection of the materials is conducted in Lower Austria. The yellow bag collection system consists of plastic packaging and metal packaging in this area. The samples were taken from the input material of a sorting plant. The samples consisted of unopened waste collection bags. The samples were taken directly after delivery to the waste sorting plant. Twelve yellow bags from separate plant deliverers were taken, and their content was the object of for the investigation in this study. All samples present a surface area of under 1.5 m² and therefore fall under the category “Films Small” as established by Van Eygen in 2018. Figure 1 gives an impression of the flexible waste plastic packaging sample. The estimated sample volume amounted to 1300 litres while the mass amounted to 39.3 kg.

### 2.2. Hand Sorting Analysis of a 2D Fraction from a Packaging Sorting Plant

The sample was examined and sorted into flexible packaging, flat and two-dimensional packaging, and bulky three-dimensional rigid packaging. The flexible waste packing samples accounted for 10.4 kg of the overall sample mass of 39.3 kg. The three-dimensional particles were discarded, and each flexible packaging was categorised further according to its usage. The investigated sample was manually sorted into these categories, and each category was weighed using a digital scale (KERN 440-49-N, precision 0.1 g). Subsequently, the flexible waste packaging was hand sorted into its respective material classes as determined by the recycling code and categorised according to their use. Figure 1 shows an example of the separated sample. On the left, the two-dimensional plastic films are depicted with the three-dimensional portion of the sample depicted on the right.

Table 1 shows part of the sample and the different categories into which the PCW has been categorised. These categories were chosen to accurately reproduce and depict the uses in which 2D materials are currently employed. Primary packaging describes material directly in contact with the product or food item respectively while secondary food packaging does not directly contact the product or food item.

### 2.3. FTIR-ATR Measurements of the Specimens

In order to classify the specimens into their respective material classes and to evaluate the proportion of multilayer material, a subset (*n* = 143) out of the complete set of samples (*N* = 842) was chosen for Fourier-Transform Infrared Spectroscopy (FTIR) in Attenuated Total Reflectance (ATR) mode.

The necessary sample size has been determined following Equation (1) while the tolerated error resulting from the sample size has been calculated using Equation (2). According to the calculation of the minimum sample size for a sample from a finite population (*N* = 843) under the premise of normal distribution (D(*z*) = 0,95; *z* = 1,96; *P* = 0.24) and a sample size of *n* = 143 randomly picked objects the tolerated error of the result equals ε = ± 6.4%, instead of ±5% for the given confidence interval.

(1) $$n\geq \frac{N}{1+\frac{\left(N-1\right)*\varepsilon^{2}}{z^{2}*P*Q}}$$

(2) $$\varepsilon =\sqrt{\frac{P*Q*z^{2}*\left(n-N\right)}{n\left(N-1\right)}}=0.064$$

In addition, the range R of values is taken as a measure of dispersion to check for comparability of the sample subset with the finite population. It is noted that some material happens to be underrepresented by weight, e.g., bags of construction and workshop material (R = 10%), generic bags (R = 7%), and wet pet food (R = 5%), and some tend to be overrepresented, e.g., packaging of bakery products (R = 9%), fresh produce (R = 4%), and dry food (R = 4%), in the sample.

The characterization was carried out using a Spectrum Two FTIR spectrometer (Perkin Elmer) equipped with a Zn/Se crystal with diamond tip in the range from 650 cm^−1^ to 4000 cm^−1^, averaging four scans per measurement point and a spectral resolution of 4 cm^−1^.

FTIR-ATR is a non-destructive method in quality assurance and inspection [13]. The subset of random objects of the total two-dimensional yellow bag sample is subjected to this further examination to identify multilayered films. The specific type of polymer can be identified by comparing the obtained infrared spectrum to literature data. Before the measurement, every sample is cleaned and cropped, and then three test points are analysed for each side of the fragment.

The materials were cleaned using a soft paper wipe and water, no solvents were used in order to not alter the samples (e.g., print removal, degradation at the surface). The samples were let dry from any water residue before performing the measurements. We ascertained that the samples were clean by visual inspection. Additionally, when performing FTIR ATR spectroscopy measurements it was possible to observe whether the materials showed additional peaks due to remaining surface contaminations or not. Preliminary trials were performed to verify that the cleaning process performed as described and to prohibit altering the samples in a way that would significantly influence the material identification.

The identified type of polymer of every specimen is noted. According to the results of these trials, the specimen with differing results for front and back are designated multilayer films. However, the FTIR-ATR characterization method allows to identify the polymeric material that is on the surface of the sample, penetrating only a few microns of the specimen’s thickness. In case of uncertainty in the assignment of a sample to the mono-material or multi-material category, additional FTIR measurements were carried out in transmittance mode to investigate the material composition through the entire thickness of the specimen, so as to ensure reliable results. The measurements in transmittance mode performed removing the ATR unit and replacing it with the proper accessory.

**Figure 2:**
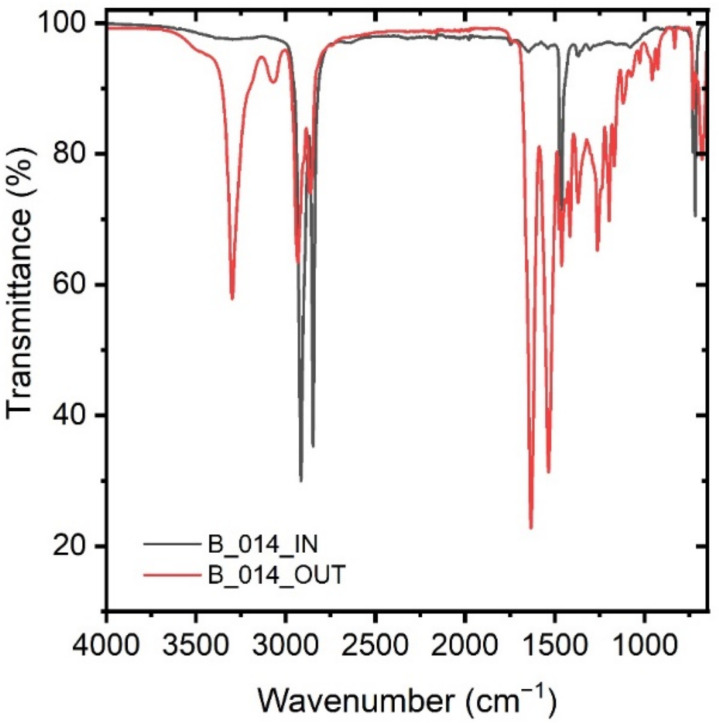
FTIR ATR spectra of a PE, PA multilayer specimen from the inner PE side (grey) and the outer PA side (red).

The spectra displayed in Figure 2 are an example of the measurements performed on the collected samples. The inner (IN) and outer (OUT) side show characteristic peaks of a polyethylene (PE) and a polyamide (PA) material, respectively. This sample was assigned to the multilayer category. The characteristic bands and their assignment can be seen in Table 2 and Table 3 below.

**Figure 3:**
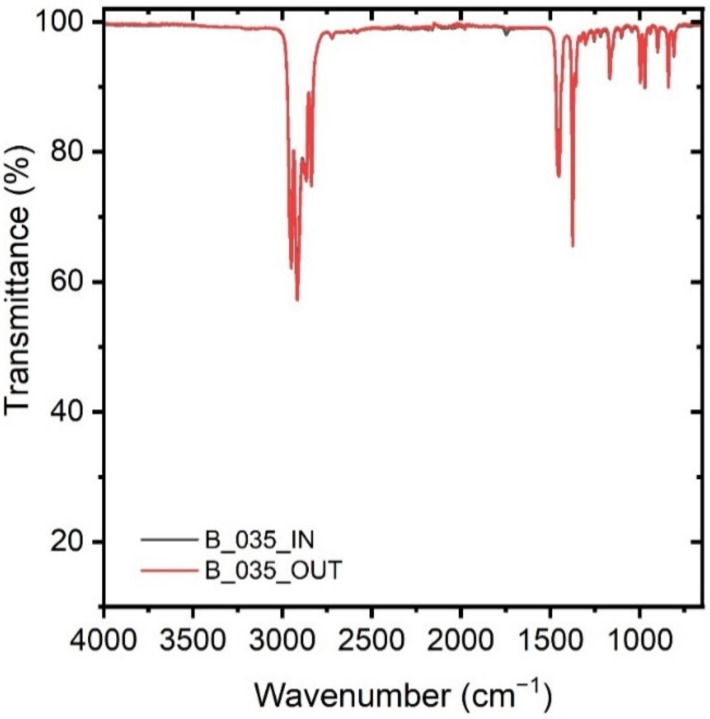
FTIR ATR spectra of a PP sample from the inner side (grey curve) and from the outer side (red curve).

The following depiction in Figure 3 is an example of the measurements performed on a collected PP sample. The inner (IN) and outer (OUT) side show characteristic peaks of a PP. This sample was assigned to the monolayer category. The characteristic bands and their assignment can be seen in Table 4 below:

### 2.4. Assessment of Recycling Potential of Mono- and Multilayer Packaging Films

This section explains how the recycling potential of multilayer films and the recycling thereof impact on the Austrian plastic packaging recycling efficiency has been estimated. The figures for the estimation are based on the material flow analysis (MFA) of the Austrian waste plastic packaging management published by Van Eygen (2018) and complemented by the hand sorting analysis findings. By supplementing the results of the hand sorting analysis with existing findings and by utilising the comparability of the two-dimensional foil fraction hand sorting analysis and the analysis carried out by Van Eygen general statements can be made for Austria [3,6,7,9,12].

The latent recycling potential of two-dimensional plastic packaging can be computed by combining the material flow of the flexible plastic packaging recycling routes with the findings of the multilayer film in the yellow bag fraction. As part of the small films, the fraction “multilayer films” takes the same recycling routes.

Three scenarios are evaluated to assess the latent recycling potential of mono- and multilayer packaging films. Each scenario assumes a different multilayer and monolayer recycling level and assesses its influence on the Austrian plastic packaging recycling efficiency. In Table 5, the three scenarios are explained in detail.

Scenario 1 reflects the current situation in Austria. Small films follow their respective recycling routes unchanged. Currently, 24 wt% (12,280 tons) of small films derived from SCW are mechanically recycled, and the remaining 76 wt% are co-incinerated. This scenario is used as the benchmark.

The current situation is a product of the presently available technology, its limitations and further of existing political and socioeconomical factors. Subsequently, substantial innovation is needed to improve the current recycling rate of two-dimensional plastic packaging. Recent reviews discussing the current technological environment have been published by Schlögl and Friedrich et al. in 2021 and 2022, respectively [16,17].

Scenario 2 assumes this substantial innovation. In this scenario, recycling techniques to detect, eject and recycle multilayer films and monolayer films are available and used on an industrial scale. This improvement facilitates the ejection of multilayer materials and leads to a pure monolayer film fraction without pollution by foreign materials introduced by missorted multilayer materials, ready for mechanical recycling and subsequent regranulation. This scenario assumes further that no material utilisation of multilayer materials is incorporated in the Austrian waste management on an industrial scale. In this scenario, multilayer materials continue to be used for energy recovery.

Scenario 3 reflects that no flexible packaging derived from SCW is used in energy recovery. New technologies to detect, eject, and recycle multilayer films are available and used on an industrial scale. Ideally, the co-incineration of monolayer- and multilayer films is entirely replaced by mechanical or chemical recycling. This scenario represents an ideal way to maximise the usage of latent potential for increasing the recycling rate by minimising the amount of thermal reuse of flexible plastic packaging.

## 3. Results and Discussion

### 3.1. Occurrence of Multilayer Material in the Waste Stream

**Figure 4:**
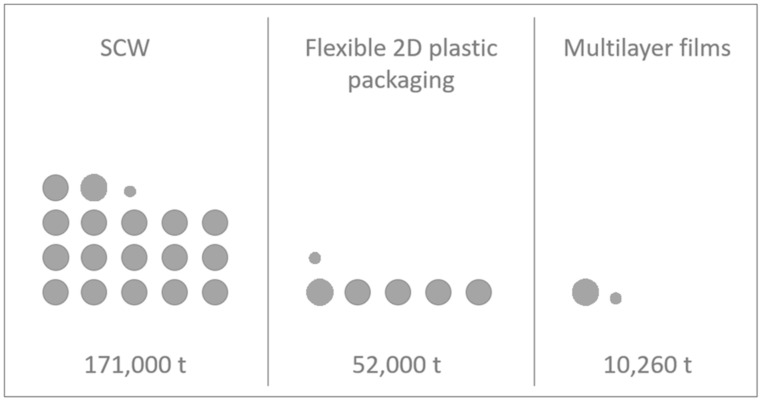
Graphical illustration of multilayer films compared to the Austrian separately collected waste (SCW) and flexible waste plastic packaging.

The hand-sorting resulted in 30 wt% of the examined SCW as flexible 2D plastic packaging. Of this fraction, two-dimensional flexible packaging with two or more layers accounted for 20 wt%. This results in 6 wt% of the total sample being multilayer materials. Extrapolating this result leads to expect that flexible 2D plastic packaging accounts for approximately 52,000 t of the 171,000 t SCW per year. This outcome is congruent with the findings of van Eygen in 2018. Supposed, the amount of multilayer packaging is also representative of the waste composition in Austria, 10,260 t or about 6 wt% of the total SCW and approximately 20 wt% of all flexible 2D plastic packaging are multilayer films. This composition is represented in Figure 4, which shows the composition of all SCW split into the categories of flexible 2D plastic packaging and the number of multilayer films. The content of multilayer films subtracted from the total amount of flexible 2D plastic packaging would yield 41,740 t of flexible monolayer packaging useable for mechanical recycling. Figure 4 is a graphical illustration of the ideal recycling potential of multilayer films compared to the Austrian SCW and flexible waste plastic packaging.

### 3.2. Material Composition of Monolayer and Multilayer Materials

**Figure 5:**
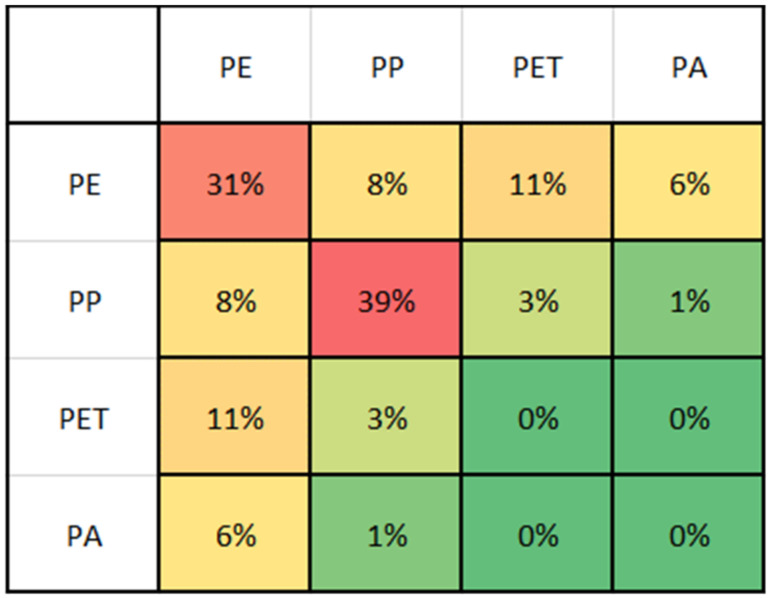
Composition of the small films according to the material class colour-coded to represent the highest proportion (**red**) to the lowest proportion (**green**).

Multilayer films tend to be numerous, lightweight, and typically accumulate in food packaging. Few but heavy specimens of multilayer films are found in product packaging. The FTIR-ATR analysis shows that multilayered material is commonly made of a combination of PE-PET, PE-PP, PE-PA, or PP-PET ranked by decreasing frequency. Other combinations, including PE-PP, PET-PP, and PET-PA, tend to be outliers. The most prevalent material found in the packaging of bakery products is PP. If the bakery packaging is multilayered, it commonly consists of PE-PA, PP-PE, or PP-PET. Meat packaging is either made of PE or a combination of PE and PET or PA. Figure 5 shows the percentage of distribution of materials in the small films fraction. The most prevalent fraction under the evaluated materials was PP with a share of 39 wt%, followed by PE monolayers with 31 wt%. Amongst the multilayer fraction, PE-PET is most abundant with 11 wt% and followed by PE-PP with 8 wt%. This composition of the small film fraction leads to the assumption that sorting processes which are able to separate monolayer from multilayer materials can both deliver a sufficient feedstock for chemical recycling to be used to recuperate functional polyolefins and PET from the waste stream and create a valuable feedstock for recycling purposes by creating clean PE and PP monolayer fractions. This approach can improve the circular economy of polymers by recuperating hitherto ignored resources for recycling by opening up a different recycling route than incineration.

### 3.3. Contribution of Monolayer and Multilayer Films in Waste Generation

The total share of multilayer films in the yellow bag is 6 wt%. Films account for about 29 wt% of all packaging evaluated. Main contributors to this are the categories primary food packaging (7.8 wt%), primary product packaging (7.9 wt%), and plastic bags (7.2 wt%), with primary packaging describing packaging directly in contact with the product.

The primary packaging groups in which multilayer films tend to accumulate are primary food packaging, where 49 wt% were multilayer films, followed by generic plastic bags, of which 34 wt% were multilayer films, and primary product packaging of which 19 wt% were multilayer films. Concerning primary food packaging, multilayer films accumulate primarily in the packaging of bakery products (16 wt%), meat (13 wt%), and dairy products (9 wt%), followed by frozen food and convenience (9 wt%), of which 16 wt%, 13 wt%, and 9 wt%, respectively, were multilayer films. These categories account for half the number of all multilayer films. Nevertheless, they only contribute about one third to the total weight of multilayer films.

**Figure 6:**
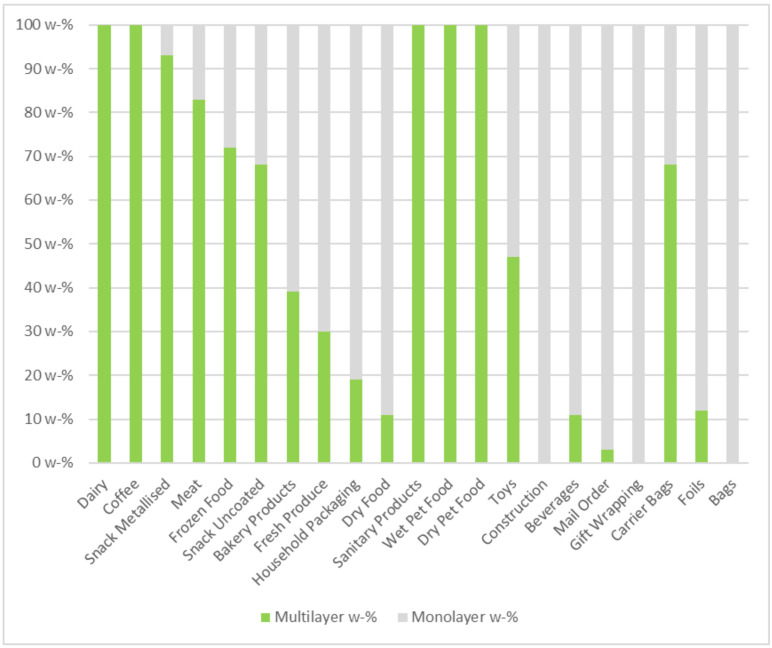
Percentage of monolayer- and multilayer materials in typical applications for small films.

Figure 6 shows the composition of the most common applications of 2D foils. It shows that the product groups where multilayer materials are most abundant are dairy, coffee packaging, snack packaging, and meat and frozen food packaging. Multilayer packaging accounts for more than 70 wt% of all packaging materials used in all of these categories. Simultaneously, no multilayer packaging is used in mail-order packaging, household product packaging, and gift wrapping.

**Figure 7:**
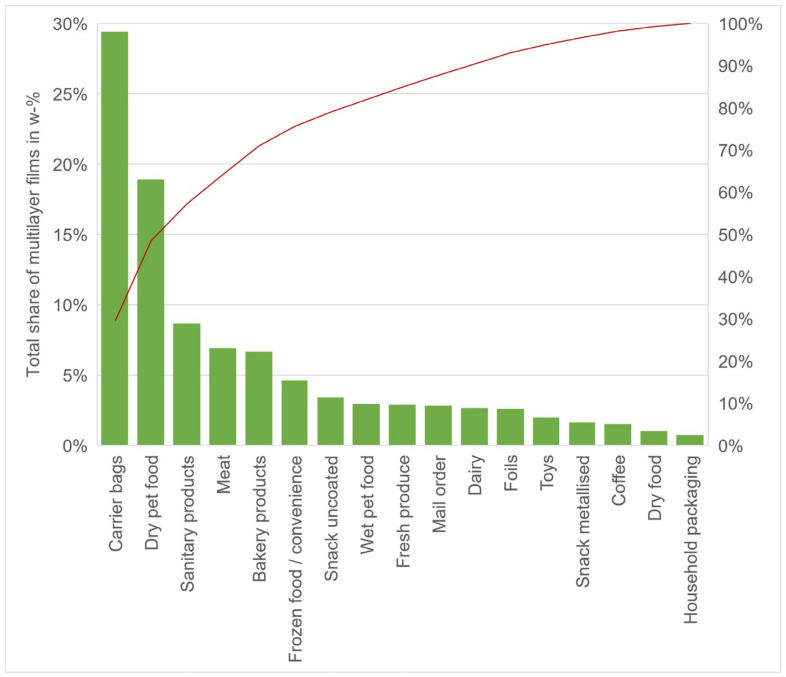
Pareto diagram depicting the share of each category to the total weight of the samples.

Figure 7 shows the distribution of multilayer films as a Pareto diagram by weight. The most common classes in which multilayer films accumulate are plastic shopping bags which account for 29 wt%. Dry pet food Packaging accounts for 19 wt%, followed by sanitary related products with 9 wt% and then by packaging for meat and bakery products with 7 wt% and convenience food with 5 wt%. These categories account for over a fifth of all multilayer films used in packaging.

### 3.4. Influence of Improved Multilayer Recycling on the Circular Economy

Based on the hand sorting analysis, the examination of the specimens with FTIR-ATR and the MFA by Van Eygen in 2018, the ideal recycling potential of films in the Austrian SCW amounts to 52,000 t [6]. Multilayer films account for 10,270 t per year in the Austrian separately waste collection. Currently, the Austrian plastic packaging recycling efficiency is 25.7 wt%. These figures show that 77,000 t of the produced 300,000 t of waste plastic packaging are recycled. The examination of three different scenarios, current situation, new technologies, and zero incineration of flexible packaging derived from SCW, evaluate the theoretical contribution of recycling of multilayer films to the Austrian plastic packaging recycling efficiency. For better understanding, each scenario is depicted as a Sankey Diagram, illustrating the mass flow of Small Films via Municipal Solid Waste (MSW), SCW, to the respective recycling technologies.

#### 3.4.1. Scenario 1: Current Situation

**Figure 8:**
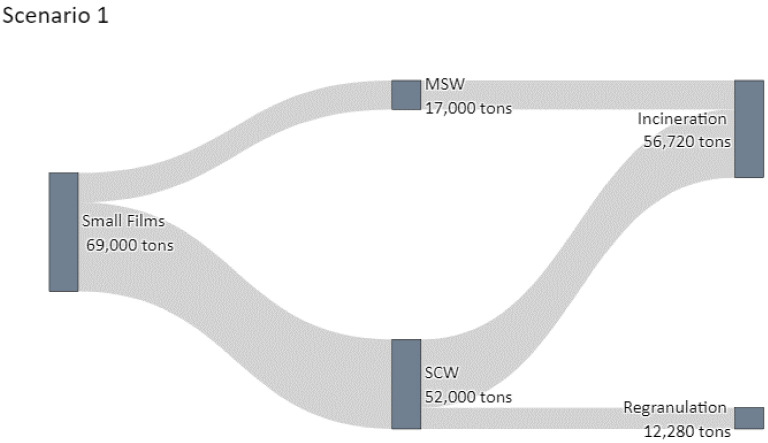
Sankey diagram depicting the waste flow in Scenario 1.

In scenario 1 the recycling routes of small films, including multilayer- and monolayer films, remain unchanged. The results of scenario one are displayed in Figure 8.

It can be seen that the bulk of the material continues to be industrially incinerated, and hardly any films are recycled. Until the development of new recycling techniques to an industrial scale, films remain subject to industrial incineration after being ejected from the mechanical recycling stream. Thus, the current recycling rate of small films to re-granulates remains at 12,280 t per year, or 18 wt%, with the overall recycling rate remaining at 25.7% [6].

#### 3.4.2. Scenario 2: New Technologies

**Figure 9:**
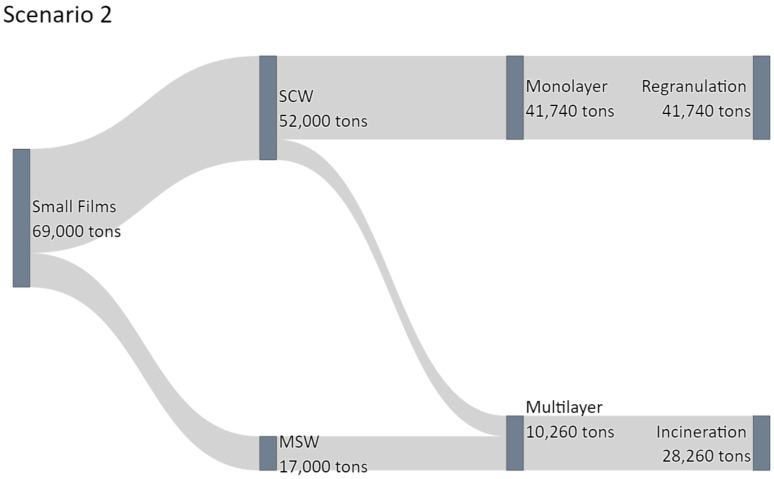
Sankey diagram depicting the waste flow in Scenario 2 with increased mechanical recycling of monolayer films.

In scenario two, the detection and ejection of multilayer films are established and complemented by a designated recycling process. Therefore, an ideal system with a successful detection and ejection rate of multilayer films of 100% can create a clean feedstock for recycling purposes. These monolayer materials have had foreign materials removed and can be incorporated into mechanical recycling processes rather than incineration. The results of scenario 2 are depicted in Figure 9.

It can be seen that of the 52,000 t of small films per year in the SCW 41,740 t are monolayer and 10,260 t of multilayers. These fractions need to be separated and recycled. By cleaning the remaining plastic packaging stream from the multilayer materials, an additional 41,740 t of monolayer materials, mainly LDPE, can be added to the recycling feedstock, increasing the recycled small films fraction from 12,280 t by 29,460 t to 41,740 t annually.

This clean material stream increases the amount of totally recycled materials from 77,000 t to 106,460 t annually, increasing the Austrian recycling quota from 25.7% to 35.5%. This approach simultaneously improves the material properties of foil recyclates and enables a broader array of applications for these recyclates. Instead of manufacturing low-grade films and foils for waste bags and agriculture, they can be incorporated into higher quality products, eliminating the necessity for adding virgin material in the production. While this approach will enable the recycling of monolayer materials, multilayer films are still subjected to thermal recovery.

#### 3.4.3. Scenario 3: Zero Incineration of Flexible Packaging Derived from SCW

**Figure 10:**
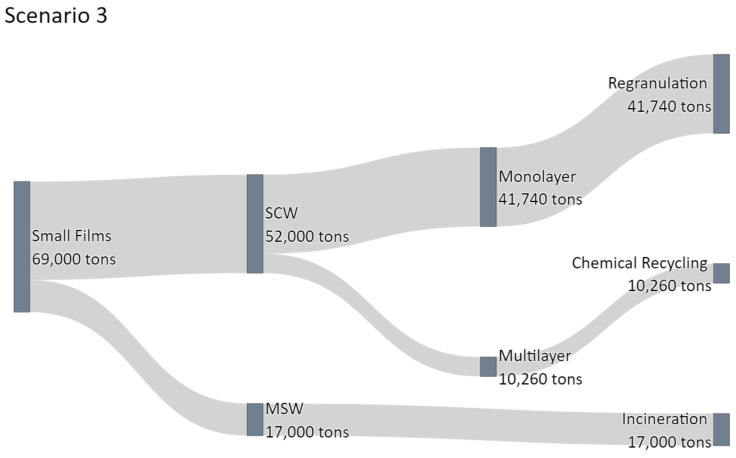
Sankey diagram depicting the waste flow in Scenario 3 with increased mechanical recycling of monolayer films and simultaneous increase in the chemical recycling of multilayer films.

Scenario 3 reflects a zero-waste approach. New technologies to detect, eject, and recycle multilayer films are available and applicable on an industrial scale. By those innovations, the previously latent recycling potential of small films can be realised by enhancing the sorting facilities of sorting systems, e.g., NIR sorting. Chemical recycling processes and solvent-based recycling processes, currently under development, have reached the market and are widely employed. This possible inclusion of mechanical and chemical recycling reduces co-incineration of monolayer- and multilayer films. The results of scenario 3 are depicted in Figure 10.

It can be seen that similar to “Scenario 2”, 41,740 t of monolayer materials can be added to the recycling feedstock. Further, the incineration of 10,260 t of multilayer films is circumvented by intensifying the mechanical recycling of monolayer films, so these ejected multilayer films can be used as value-adding feedstock for chemical recycling processes.

This inclusion of multilayer films increases the amount of recycled small films fraction from 12,280 t to 52,000 t by 39,720 t annually. This change in recycling methods increases the amount of totally recycled materials from 77,000 t to 116,720 t annually, increasing the Austrian recycling quota from 25.7% to 38.9%. The enhanced separation of the small films fraction creates a value-adding pool of resources, improves the circular economy of plastics and at the same time creates a feedstock for future chemical recycling by providing a multilayer fraction, which would otherwise have been incinerated.

## 4. Discussion

With 69,000 t annually, small films represent a sizeable portion of the total waste generation in Austria each year. Despite extensive use in packaging, these materials are not commonly mechanically recycled but primarily used in thermal energy recovery or as residue derived fuel in industrial co-incineration. This current state of affairs leads to diminished recycling rates and wasted potential for improving the circular economy of polymers and increases the number of virgin materials needed for production each year to satisfy the growing interest in packaging. As a packaging material, multilayer films are most abundant in the packaging of meat and dairy and the packaging of sanitary products and large carrier bags. Currently, the waste stream incorporates approximately 30 wt% flexible 2D plastic packaging of the 171,000 t separately collected waste (SCW) per year in Austria. The hand-sorting showed that 6 wt% of the examined materials were multilayer films. Extrapolating these results leads to expect that about 10,260 t of the total SCW are multilayer films, of which the most common types of polymers used in multilayer materials are PE and PET, which further account for 10% of all 2D plastic packaging evaluated.

Based upon these results, which were correlated with the findings in existing studies, three scenarios showing the latent potential in small film recycling were evaluated. These scenarios show the potential for increasing the recycling quota in Austria, and subsequently, the reaching of the recycling goals set by the EU was evaluated. It has been shown that the introduction of improved methods for separating multilayer- and monolayer materials leading to a clean material stream of monolayer materials uninhibited by foreign materials introduced by multilayer packaging can be used in mechanical recycling. This approach relieves the thermal recovery plants by producing a feedstock of clean monolayer materials of 41,740 t per year for mechanical recycling, yielding an increase in recycling quota to 35.5% from the current rate of 25.7%, as shown in Table 6. With the introduction of chemical recycling and solvent-based recycling to separate multilayer material compounds, polymers’ recovery is likely to increase further. This fraction encompasses a reliable feedstock with an annual consumption of multilayer materials accounting for 6 wt% of the SCW collected each year. The introduction of chemical recycling processes in the Austrian waste management system to decompose multilayer materials can further increase the annual recycling quota to 38.9%, reducing the number of incinerated polymers.

**Figure 11:**
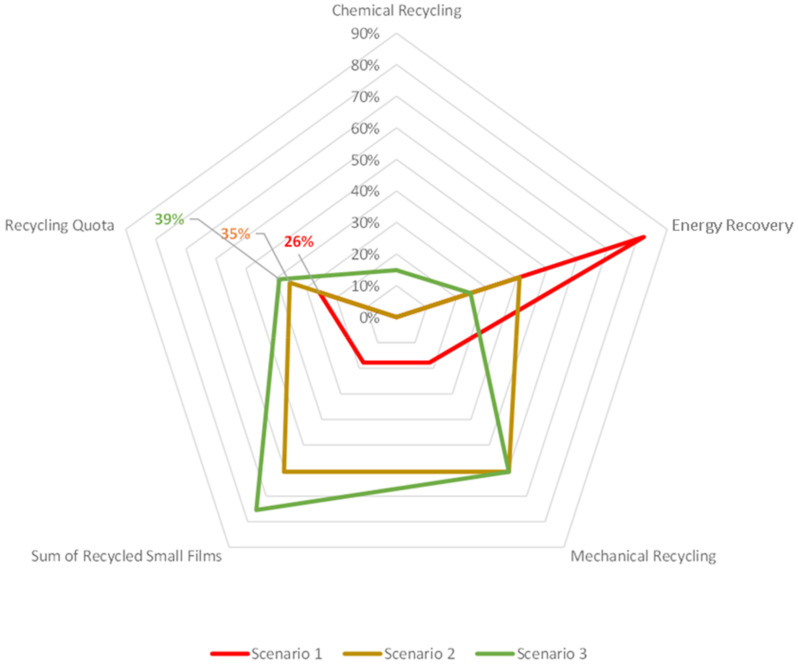
Radar diagram comparing the three evaluated scenarios based on the characteristic metrics: Energy recovery, Mechanical recycling, sum of recycled films, recycling quota and chemical recycling.

Figure 11 shows a radar diagram comparing the three evaluated scenarios based on the characteristic metrics. These metrics are the recycling quota, the quota of recycled small films and the percentages for each recycling method. It can be seen that the ratio of thermal recycled small films progressively decreases from Scenario 1 to Scenario 3. Scenario 3 shows the least incinerated small films, which yields the highest recycling quota.

While the depicted scenario is confined to information gathered from Austrian waste processing plants, similar waste composition research has been performed in other European countries. An especially relevant and recent survey by Schmidt et al. who surveyed the recycling of two-dimensional waste reported similar composition of 2D waste in Germany [18]. This implies that the results may be applicable to the German waste management sector, but further research is needed, especially to predict the implications on the waste management in countries who deviate substantially in respect to culture and industrialisation from the current situation in Austria as these differences makes it difficult to predict the effect these changes might have on other countries around the world.

## Figures and Tables

**Table 1:** Definition of sorting categories of application of two-dimensional plastic waste.

Category 1	Category 2	Example/Packaging of:
Primary food packaging	Bakery products Coffee Dairy Dry food Fresh produce Frozen food/convenience Household packaging Meat Snack metallised Snack uncoated	Bread, rolls, pastry Coffee bags Sliced cheese, yoghurt lids Rice, noodles, cereal Fruit and vegetables Frozen vegetables, Zip-bags, cling film Sausages, steak, cold cuts Chocolate Bars, Granola Bars
Secondary food packaging	Beverage	Wrapping of six-packs
Primary product packaging	Construction/workshop Dry pet food Garden Household products Sanitary products Toys Wet pet food	Cement, Tools, oil Soil, mulch, bark chips, Clothes, toner Toilet paper, kitchen roll interlocking (plastic) brick
Secondary product packaging	Gift wrapping	Wrapping paper, ribbons,
Bags	Generic bags	Transparent single-use bags
Foils	Generic foils	Pieces of foil

**Table 2:** FTIR ATR characteristic bands of PE and their assignment [13,14,15].

Wavenumber [cm^−1^]	Comment
2914, 2850	Stretching vibrations of CH_2_
1471	Bending vibrations of CH_2_
717	Rocking vibrations of CH_2_

**Table 3:** FTIR ATR characteristic bands of polyamide and their assignment [13,14,15].

Wavenumber [cm^−1^]	Comment
3295, 3070	Stretching vibration of NH
3000–2840	Stretching vibrations of CH_2_
1633	Vibration of C=O
1534	Bending vibration of NH, stretching vibration of CN
1462	Bending vibration of CH_2_
1368	Deformation vibration of CH_2_
1260	Bending vibration of NH, stretching vibration of CN
730	Rocking vibrations of CH_2_

**Table 4:** FTIR ATR characteristic bands of polypropylene and their assignment [13,14,15].

Wavenumber [cm^−^^1^]	Comment
2959, 2916, 2868, 2837	Stretching vibrations (symmetrical and asymmetrical) of CH_2_ and CH_3_
1452, 1376	Bending vibrations of CH_2_ and CH_3_
1167	Rocking vibrations of CH_3_, bending vibrations of CH, stretching vibrations of C-C
998,	Rocking vibrations of CH_3_, bending vibrations of CH_3_, bending vibrations of CH
972	Stretching vibration of C-C, rocking vibrations of CH_3_
841, 732	Rocking vibration of CH_2_, stretching vibrations of C– CH_3_
809	Rocking vibration of CH_2_, stretching vibrations of C-C, stretching vibrations of C-CH

**Table 5:** Different scenarios for the assessment of the contribution of multilayer films to the Austrian plastic packaging recycling-quota.

	Scenario 1		Scenario 2		Scenario 3	
	Monolayer	Multilayer	Monolayer	Multilayer	Monolayer	Multilayer
Recycling	24 wt%		100 wt%		100 wt%	100 wt%
Incineration	76 wt%			100 wt%		

**Table 6:** Results of the critical factors of all evaluated scenarios in tabular form.

Scenario	Scenario 1: Current Situation	Scenario 2: Mechanical Recycling of Monolayer Films Derived from SCW	Scenario 3: Zero Incineration of Flexible Packaging Derived from SCW
Total Amount of Waste	300,000 t	300,000 t	300,000 t
Sum of Small Films	69,000 t	69,000 t	69,000 t
Chemical/Solvent-Based Recycling	0 t	0 t	10,260 t
Energy Recovery	56,720 t	28,260 t	17,000 t
Mechanical Recycling	12,280 t	41,740 t	41,740 t
Sum of Recycled Small Films	12,280 t	41,740 t	52,000 t
Total Recycling	77,000 t	106,460 t	116,720 t
Recycling Quota	25.7%	35.5%	38.9%

## Data Availability

The data presented in this study are available on request from the corresponding author.
